# Polyphenol consumption and Nonalcoholic fatty liver disease risk in adults

**DOI:** 10.1038/s41598-024-57416-0

**Published:** 2024-03-21

**Authors:** Mehran Rahimlou, Ghazal Baghdadi, Ali Khodi, Zahra Rahimi, Nader Saki, Nasrin Banaei Jahromi, Bahman Cheraghian, Ronia Tavasolian, Seyed Ahmad Hosseini

**Affiliations:** 1 Department of Nutrition, School of Public Health, Zanjan University of Medical Sciences, Zanjan, Iran; 2 Department of Nutrition, School of Public Health, Iran University of Medical Sciences, Tehran, Iran; 3 Taylor’s University, Subang Jaya, Malaysia; 4 Department of Biostatistics and Epidemiology, School of Public Health, Ahvaz Jundishapur University of Medical Sciences, Ahvaz, Iran; 5 Department of Otolaryngology, Head and Neck Surgery, Hearing Research Center, Ahvaz Jundishapur University of Medical Sciences, Ahvaz, Iran; 6 Department of Nutrition, School of Allied Medical Sciences, Ahvaz Jundishapur University of Medical Sciences, Ahvaz, Iran; 7 Nutrition and Metabolic Disease Research Center, Clinical Sciences Research Institute, Ahvaz Jundishapur University of Medical Sciences, Ahvaz, Iran; 8 Alimentary Tract Research Center, Department of Biostatistics & Epidemiology, School of Public Health, Ahvaz Jundishapur University of Medical Sciences, Ahvaz, Iran

**Keywords:** Polyphenol, Flavonoid, Phenolic acid, Lignin, NAFLD, Cohort, Endocrine system and metabolic diseases, Endocrinology, Gastroenterology

## Abstract

In this cross-sectional investigation, the primary objective was to explore the correlation between the consumption of polyphenols and the likelihood of non-alcoholic fatty liver disease (NAFLD) in the adult population participating in the Hoveyzeh cohort. Data from the Hoveyzeh cohort study, part of the Persian Cohort Study, involving 10,009 adults aged 35–70, were analyzed. Exclusions were made for missing data, extreme energy intake, and liver cancer patients. Dietary habits were assessed using a food frequency questionnaire, and polyphenol intake was calculated using the Phenol Explorer database. Logistic regression analyses, adjusted for confounders, were performed to assess the relationship between polyphenol subclasses (total polyphenols, total flavonoids, phenolic acid, and lignin) and NAFLD. Among 9894 participants, those in the highest quintile of total polyphenol (OR 0.65, CI 0.5–0.84; *P* = 0.007), phenolic acid (OR 0.67, CI 0.52–0.86; *P* < 0.001), and lignin intake (OR 0.69, CI 0.52–0.87; *P* = 0.001) demonstrated lower odds of NAFLD compared to the lowest quintile, even after adjusting for confounding factors. However, no significant association was found between total flavonoid intake and NAFLD (OR 1.26, CI 0.96–1.67; *P* = 0.47). Subgroup analysis indicated a significant inverse association between total polyphenols and NAFLD in women (OR 0.64, CI 0.42–0.93; *P* = 0.001). Higher intake of total polyphenols, phenolic acid, and lignin was associated with reduced odds of NAFLD among adults in the Hoveyzeh cohort. This suggests that dietary patterns rich in these polyphenols may play a role in mitigating the risk of NAFLD. Further interventional and longitudinal studies are needed to validate these findings and explore potential preventive strategies involving polyphenol-rich diets.

## Introduction

NAFLD is a medical problem predominantly marked by the gathering of fat in the liver tissue, specifically exceeding 5% of hepatocytes^1^. The impact of NAFLD is reflected in its range of pathological manifestations, spanning from Simple NAFLD to steatohepatitis (Non-Alcoholic Steatohepatitis—NASH), ultimately culminating in hepatic cirrhosis and hepatocarcinoma^2,3^. Increased mortality is linked to the advanced phases of NAFLD. However, it is important to note that all stages of NAFLD pose a considerable risk for other chronic diseases. Its definition encompasses a state wherein the accumulation of fat in the liver tissue exceeds the typical amount and exhibits histopathological features akin to alcohol-induced liver steatosis. Notably, this condition is identified in individuals who consume minimal to no alcohol^4–7^.

Approximations suggest that the global incidence of NAFLD varies between 20 and 30%^8^. Based on Adibi et al. study, Prevalence of NAFLD among the Iranian adults was 39.3%^9^.Insulin resistance, obesity and metabolic syndrome are recognized as prominent risk factors linked to NAFLD^10^. The widespread occurrence of NAFLD diagnosis globally is closely connected to the prevalent adoption of sedentary lifestyles coupled with excessive caloric intake. Implementing changes in lifestyle, particularly achieving weight reduction, stands out as the most effective therapeutic approach for managing NAFLD. A weight loss of at least 7 to 9% is necessary to alleviate hepatic inflammation^11–14^. Moreover, aside from overall energy consumption, dietary composition also plays a role in influencing metabolic functions. Recent research indicates that incorporating diets rich in polyphenols may be beneficial in preventing and treating chronic conditions, such as obesity, some types of the cancer, diabetes, and other chronic diseases^15–18^.

Polyphenols are naturally occurring phytochemical compounds found in plant-based foods, encompassing fruits, vegetables, whole grains, and beverages such as coffee, tea, red wine, and dark chocolate^6,17,19,20^. Polyphenols are the predominant antioxidants present in diet, and their impacts, akin to those of vitamins, form the fundamental basis for the well-established health benefits traditionally attributed to fruits and vegetables across various diseases^21–23^. Moreover, it has been documented that these substances could influence de novo lipogenesis ^21,24^.

Nevertheless, there is a scarcity of experimental or epidemiological studies exploring the protective effects of these components in NAFLD. The majority of the research has concentrated on specific categories of polyphenols and flavonoids, with less emphasis on assessing the intake of polyphenol subclasses^25,26^. Therefore, this population bases study was aimed to investigate the relationship between the consumption of total polyphenols, total flavonoids, phenolic acid and lignin with the odds of NAFLD among the adults.

## Subjects and methods

### Study design and population

This cross-sectional investigation utilized data derived from the initial phase of the national Persian Cohort, specifically the Hoveyzeh cohort study (HCS). The HCS, conducted from May 2016 to August 2018, is a prospective study focused on non-communicable diseases within the Arab population. A total of 10,009 adults aged 35–70 participated in Hoveyzeh and Rofayyeh cities, located in the Khuzestan province of Southwest Iran. Prior to collecting data, participants were provided with a detailed explanation of the study’s objectives and methods, and subsequently informed consent was obtained from all subjects.

The HCS cohort study is an integral part of the Prospective Epidemiological Research Studies in Iran, known as the PERSIAN Cohort Study, and has received approval from the Ethics Committees in the Ministry of Health and Medical Education, as well as the Digestive Diseases Research Institute at Tehran University of Medical Sciences in Iran. Further information about this study has been previously published elsewhere^27,28^. For this study, we excluded people who had missing data, people whose daily energy intake was higher than 4500 kcal or less than 700 kcal, and also patients with liver cancer, patients who were taking hepatotoxic drugs or had followed restricted diets during the last three months (n = 115) and final analysis was done on data from 9894 adults. The research received ethical approval from the Ethics Committee of Ahvaz Jundishapur University of Medical Sciences under the reference number (IR.AJUMS.REC.1398.640).

### Anthropometric measurements

Digital scales (Seca 755) were employed to measure weight with an accuracy of 100 g, while individuals wore lightweight clothing and no shoes. Height, accurate to 0.5 cm, was measured using a Seca stadiometer (Seca company, Model 206, Germany) with individuals standing in a normal alignment, shoulders relaxed, and without shoes. Body mass index (BMI) was computed by dividing weight (in kilograms) by the square of height (in meters).

### Assessment of blood pressure and biochemical markers

Following a fasting period of 10 to 12 h, blood specimens were gathered from each participant. To isolate serum from the blood, the samples underwent centrifugation for 15 min at 1000 revolutions per minute and were subsequently preserved at -80 °C until analysis. Additional procedural information can be found in the preceding research^28^. The glucose oxidase method was employed to measure fasting blood glucose (FBG). Enzymatic kits from Pars Azmoon, Iran, were utilized to determine total cholesterol (TC), triglyceride (TG), aspartate aminotransferase (AST), alanine transaminase (ALT), and high-density lipoprotein (HDL). Systolic and diastolic blood pressure were recorded twice for each arm using Riester sphygmomanometers, separated by a 10-min interval, and the average of the two measurements was reported. The diagnosis of NAFLD was based on the patient's medical record and the reports of the individual's family physician in the patient's medical record.

### Dietary assessment

A semi-quantitative food frequency questionnaire (FFQ) comprising 130 food items was employed to evaluate the typical dietary habits of individuals^28^. Participants were instructed to indicate the frequency of consuming each item over the past year, specifying whether it occurred daily, weekly, monthly, or annually. Trained dietitians conducted face-to-face interviews with participants to complete all questionnaires. The quantity of each food item was converted into grams per day using household measures. Subsequently, the recorded food intake in grams per day was entered into Nutritionist IV to compute the overall energy and nutrient intake.

### Polyphenol evaluation

The assessment of polyphenol consumption in the diet involved the utilization of an enhanced edition of the phenol explorer database (www.phenol-explorer.eu). This database encompasses 501 polyphenols categorized into 6 classes and 31 subclasses. Additionally, it provides insights into the impact of food processing on the levels of polyphenols ^29^. We gathered information on the total polyphenol content, along with its primary subcategories, including flavonoids, phenolic acids, and lignans, from a selection of 80 food items. The overall polyphenol content was predominantly determined using the Folin assay. In cases where this method was not applicable, we aggregated the individual polyphenol subclasses to derive the total polyphenol content. Regarding flavonoids and phenolic acids, we computed their concentrations through chromatography following hydrolysis whenever applicable, and lignans measured using chromatography after hydrolysis. In all computations, we took into account food preparation techniques like cooking, frying, and other methods, incorporating the retention factor as documented in the "phenol explorer database." Subsequently, the quantities of polyphenols were assessed by calculating the polyphenol content in conjunction with the daily intake of each food item.

### Statistical analysis

Statistical analysis was performed utilizing the Statistical Package for the Social Sciences (SPSS) software, version 21 (developed by SPSS Inc. in Chicago, IL, USA). The normality of the data was assessed using the Kolmogorov–Smirnov test and a histogram chart. Baseline characteristics and dietary intakes were presented as either mean ± standard error (SE) for quantitative variables, and as numbers and percentages for qualitative variables. The examination of continuous and categorical variables was conducted using either the Analysis of Variance (ANOVA) test or the Chi-square test across the dietary polyphenol intake. Ultimately, logistic regression analyses were conducted, and the reported outcomes included the level of significance, odds ratio (OR), and 95% confidence interval (CI). A statistical significance level was considered achieved when *P* < 0.05. The regression analyses were adjusted for confounding variables that could impact the association between the dietary polyphenols and NAFLD. These variables comprised age, energy intake, gender, smoking, alcohol consumption, physical activity levels, BMI, and education level.

### Ethics approval and consent to participate

The study was carried out in accordance with the Declaration of Helsinki, and the research protocol received approval from the of Ahvaz Jundishapur University of Medical Sciences under the reference number (IR.AJUMS.REC.1398.640). Prior to collecting data, participants were provided with a detailed explanation of the study's objectives and methods, and subsequently informed consent was obtained from all subjects.

## Results

The data obtained from 9894 adults participating in the Hoveyzeh cohort were participated in the current investigation. Table 1 summarized the demographic, anthropometric and clinical characteristics of people based on quintiles of polyphenol intake. The mean age of the of the study participants was 48.3 (0.22) years, and we found a significant difference in the age of the participants among the quintiles of dietary polyphenol intake (*P* < 0.001). Also, 3931 participants were male and the rest were female, and in terms of gender distribution, a significant difference was seen between the quintiles of dietary polyphenol intake (*P* < 0.001). In term of smoking and alcohol intake, there wasn’t any significance difference between subjects on quintiles of polyphenol intake (*P* = 0.13 for smoking and *P* = 0.204 for alcohol consumption). Moreover, we found a significant differences between participants in different quintiles of polyphenol intake in term of BMI distribution (*P* = 0.003), blood glucose (*P* < 0.001) and triglyceride (*P* < 0.001). NAFLD was identified in 1134 (prevalence, 14.52%) of the 9894 subjects. The findings from the investigation indicated that the prevalence of NAFLD in people who were in the upper quintile of dietary intake of polyphenols was significantly lower than in people who were in the lower quintile (254 patients vs. 312 patients; *P* = 0.02).

**Table 1:** Characteristics of participants according to quintiles of the dietary polyphenol intake.

Dietary polyphenol intake						
	Quintile1	Quintile2	Quintile3	Quintile4	Quintile5	*P* value ^a^
	(n = 1979)	(n = 1978)	(n = 1980)	(n = 1979)	(1978)	
Mean total polyphenol intake	2435.13	3358.87	4099.51	5039.87	7891.70	< 0.001
Age^b^	50.83 (0.22)	49.18 (0.21)	48.74 (0.20)	47.61 (0.19)	47.53 (0.19)	< 0.001
Gender, n (%)						
Men	427 (21.6)	592 (29.9)	805 (40.7)	1007 (50.9)	1100 (55.6)	< 0.001
Women	1552 (78.4)	1386 (70.1)	1175 (59.3)	972 (49.1)	878 (44.4)	
Education, n (%)						
Illiterate	1346 (68)	1291 (65.3)	1223 (61.8)	1118 (56.5)	1167 (59)	
Primary	283 (14.3)	308 (15.6)	339 (17.1)	340 (17.2)	368 (18.6)	< 0.001
Secondary	93 (4.7)	112 (5.7)	133 (6.7)	166 (8.4)	159 (8)	
High	127 (6.4)	138 (7)	138 (7)	182 (9.2)	147 (7.4)	
University	130 (6.6)	129 (6.5)	147 (7.4)	173 (8.7)	137 (6.9)	
Physical activity level, n (%)						
Inactive	561 (28.3)	509 (25.7)	502 (25.4)	455 (23)	454 (23)	
Low	584 (29.5)	507 (25.6)	524 (26.5)	495 (25)	384 (19.4)	< 0.001
Moderate	489 (24.7)	532 (26.9)	480 (24.2)	486 (24.6)	495 (25)	
Active	345 (17.4)	430 (21.7)	474 (23.9)	543 (27.4)	645 (32.6)	
Smoking, n (%)						
Yes	288 (14.6)	309 (15.6)	327 (16.5)	306 (15.46)	290 (14.66)	0.13
Alcohol consumption, n (%)						
Yes	31 (1.5)	29 (1.5)	33 (1.7)	37 (1.9)	33 (1.67)	0.204
Body Mass Index, n (%)						
Underweight (< 18.5)	37 (1.9)	35 (1.8)	29 (1.5)	24 (1.2)	24 (1.2)	
Normal (18.5–24.9)	483 (24.4)	424 (21.4)	421 (21.3)	435 (22)	458 (23.2)	0.003
Overweight (25–29.9)	669 (33.8)	731 (37)	734 (37.1)	733 (37)	793 (40.1)	
Obesity (> 30)	790 (39.9)	788 (39.8)	796 (40.2)	787 (39.8)	703 (35.5)	
Blood glucose (mg/dl)^b^	119.76 (1.30)	112.34 (1.11)	113.00 (1.22)	110.51 (1.08)	107.58 (0.97)	< 0.001
Triglycerides (mg/dl)^b^	172.88 (2.74)	167.55 (2.39)	162.36 (2.29)	158.82 (2.50)	156.99 (2.14)	< 0.001
Total cholesterol (mg/dl)^b^	190.61 (0.90)	189.49 (0.90)	188.49 (0.92)	189.30 (0.90)	190.61 (0.90)	0.57
High density lipoprotein (mg/dl)^b^	50.32 (0.26)	50.45 (0.27)	50.00 (0.26)	49.57 (0.27)	50.82 (0.28)	0.07
AST (mg/dl)^b^	19.56 (0.20)	19.18 (0.21)	18.62 (0.24)	18.12 (0.18)	18.00 (0.20)	< 0.001
ALT (mg/dl)^b^	24.07 (0.37)	23.21 (0.39)	21.27 (0.32)	19.71 (0.28)	18.89 (0.31)	< 0.001
NAFLD, n (%)						
Yes	312 (15.76)	307 (15.52)	291 (14.69)	273 (13.79)	254 (12.84)	0.02

^a^*P*-values were determined using analysis of variance (ANOVA) test for continuous variables and Chi-square test for categorical variables.

^b^Data are given as means, with standard error (SE) in parentheses.

Nutrient and food consumption according to quintiles of the dietary polyphenol intake among the participants showed in Table 2. The mean energy intake of people in the highest quintile of dietary polyphenol intake was significantly higher than in the first quintile (3376.9 kcal/day vs. 2182.5 kcal/day; *P* < 0.001). Also, participants in the higher quantile of polyphenol intake, consumed significantly higher amounts of carbohydrate (*P* = 0.02), total fat (*P* = 0.01), PUFA (*P* = 0.03), fruits (*P* = 0.02) and vegetables (*P* = 0.04) and lower amounts of red meat (*P* = 0.03) than subjects in the lower quantile of dietary polyphenol intake. We couldn’t find any significant differences between participants in term of other macro and micronutrients (*P* > 0.05).

**Table 2:** Nutrient and food consumption according to quintiles of the dietary polyphenol intake.

Dietary polyphenol intake						
	Quintile1	Quintile2	Quintile3	Quintile4	Quintile5	*P* value^a^
	Mean (95% CI)	Mean (95% CI)	Mean (95% CI)	Mean (95% CI)	Mean (95% CI)	
Energy intake (kcal/d)^b^	2182.5 (2158.0, 2206.9)	2731.9 (2705.4, 2758.5)	3073.2 (3044.0, 3102.5)	3187.8 (3064.6, 3274.9)	3376.9 (3236.1, 3485.2)	< 0.001
Diet inflammatory index	1.30 (1.2, 1.3)	0.21 (0.13, 0.28)	− 0.39 (− 0.44, − 0.31)	− 0.94 (− 1.02, − 0.87)	− 1.17 (− 1.24, − 1.09)	< 0.001
Carbohydrate intake (g/d)	513.2 (498.3, 523.3)	527.1 (517.7, 530.6)	531.0 (523.8, 538.2)	534.4 (527.0, 541.8)	546.6 (519.2, 553.9)	0.02
Protein intake (g/d)	96.32 (95.0, 97.6)	97.12 (95.7–98.4)	96.00 (94.7, 97.2)	96.87 (95.5, 98.1)	98.62 (94.3, 99.9)	0.48
Total fat intake (g/d)	64.44 (62.1, 65.7)	67.83 (66.5, 69.1)	69.66 (67.4, 70.3)	71.4 (69.5–73.6)	73.9 (71.8–74.9)	0.01
Cholesterol intake (g/d)	235.4 (229.7, 241.2)	236.6 (230.7, 242.4)	234.2 (228.7, 239.6)	236.8 (231.2, 242.5)	238.4 (222.6, 244.3)	0.23
Saturated fats (g/d)	23.9 (22.5, 24.8)	25.38 (24.7, 26.0)	24.71 (24.0, 25.3)	25.42 (24.7, 26.1)	26.35 (23.6, 27.0)	0.11
Polyunsaturated fats (g/d)	13.28 (13.1, 13.6)	13.41 (13.18, 13.6)	13.68 (13.4, 13.9)	13.71 (13.4, 13.9)	13.82 (13.5, 14.0)	0.03
Monounsaturated fats (g/d)	19.11 (18.9, 19.9)	20.41 (20.0, 20.8)	20.13 (19.7, 20.5)	20.45 (19.8, 20.7)	21.11 (20.7, 21.5)	0.02
Fiber intake (g/d)	33.03 (32.5, 33.5)	33.78 (33.2, 34.2)	35.77 (33.2, 36.2)	35.91 (34.0, 36.8)	36.25 (34.8, 37.5)	0.19
Vegetables (g/d)	569.8 (562.3, 585.2)	579.2 (567.2, 591.1)	589.2 (577.5, 601.0)	591.3 (578.8, 603.7)	598.4 (582.1, 606.7)	0.04
Fruits (g/d)	439.1 (428.0, 450.2)	446.5 (434.8, 458.1)	454.2 (442.5, 465.8)	462.0 (449.4, 471.6)	469.6 (457.8, 480.4)	0.02
Legumes (g/d)	36.91 (35.6, 38.1)	36.44 (35.2, 37.6)	37.78 (36.5, 39.0)	37.52 (35.3, 38.7)	38.54 (35.3, 39.4)	0.51
Dairy products (g/d)	292.5 (280.9, 304.0)	295.8 (283.36, 308.2)	301.0 (289.3, 312.6)	301.2 (288.3, 314.0)	305.1 (293.1, 317.0)	0.62
Fish and sea foods (g/d)	32.45 (31.3, 33.5)	33.21 (32.0, 34.4)	32.84 (31.6, 33.9)	31.98 (30.8, 33.1)	32.99 (31.8, 34.1)	0.60
Red meat (g/d)	17.03 (16.2, 17.7)	17.15 (16.4, 17.8)	16.96 (16.2, 17.6)	16.71 (16.0, 17.4)	15.69 (14.9, 16.3)	0.03
Refined grain (g/d)	689.9 (678.6, 701.2)	693.7 (682.1, 705.2)	684.5 (673.6, 695.5)	696.8 (685.4, 708.3)	691.2 (679.7, 700.4)	0.29
Sodium intake (mg/day)	3489.2 (3362.1, 3549.4)	3412.4 (3353.6, 3567.8)	3418.4 (3321.3, 3492.6)	3292.16 (3185.2, 3352.4)	3323.4 (3196.2, 3425.6)	0.13
Potassium intake (mg/day)	3260.17 (3165.4, 3319.2)	3319.5 (3244.3, 3429.4)	3265.3 (3173.2, 3332.3)	3343.2 (3221.3, 3422.3)	3321.3 (3266.3, 3419.4)	0.26
Calcium intake (mg/day)	923.46 (856.4, 984.7)	942.3 (860.3, 973.4)	968.2 (903.2, 1050.3)	958.32 (932.1, 967.3)	964.3 (948.2, 984.3)	0.28

^a^*P*-values were determined using ANOVA test.

^b^Data are given as means, with 95% CI in parentheses.

Table 3 showed the association between total and special polyphenols with risk of NAFLD. In the crude model and in term of total polyphenols, we found a significant inverse correlation between amount of dietary polyphenols intake and odds of NAFLD (OR 0.65, CI 0.5–0.84; *P* = 0.007). Also, after adjusting for confounding factors, in the full adjusted model, again, participants in the higher quantile of total polyphenols had a significant lower odd of NAFLD compared the reference group (OR 0.65, CI 0.5–0.84; *P* = 0.007). Also, in the subgroup analysis, only in women, we found a significant inverse association between total polyphenols intake and odds of NAFLD (OR 0.64, CI 0.42–0.93; *P* = 0.001).

**Table 3:** Odds ratio (OR) and 95% confidence intervals (CI) for the relation between the dietary polyphenol subgroups intake and NAFLD.

Variable	Variable in Quintile					*P* for trend
	Quintile1	Quintile2	Quintile3	Quintile4	Quintile5	
Total polyphenols (mg/day)	(< 2993.14)	(2993.14–3714.8)	(3714.8–4510.7)	(4510.7–5730.1)	> 5730.1	
OR^1^ (CI)	Ref	0.88 (0.69–1.12)	0.88 (0.70–1.12)	0.83 (0.65–1.06)	0.65 (0.50–0.84)	0.007
OR^2^ (CI)	Ref	0.99 (0.74–1.32)	0.95 (0.73–1.24)	0.86 (0.70–1.16)	0.72 (0.57–95)	0.02
Men						
OR^2^ (CI)	Ref	1.67 (0.94–2.94)	1.12 (0.61–2.06)	0.97 (0.73–1.37)	0.89 (0.70–1.54)	0.25
Women						
OR^2^ (CI)	Ref	0.95 (0.68–1.34)	0.86 (0.64–1.14)	0.78 (0.57–1.06)	0.64 (0.42–0.93)	0.001
Total Flavonoids (mg/day)	(< 145.5)	(145.57–197.6)	(197.69–258.4)	(258.48–350.90)	(> 350.9)	
OR^1^ (CI)	Ref	1.29 (1.30–2.20)	1.60 (1.23–2.09)	1.43 (1.10–1.88)	1.26 (0.96–1.67)	0.47
OR^2^ (CI)	Ref	1.69 (1.29–2.21)	1.67 (1.26–2.20)	1.47 (1.10–1.96)	1.35 (0.99–1.87)	0.31
Men						
OR^2^ (CI)	Ref	1.39 (0.80–2.41)	1.48 (0.87–2.51)	1.29 (0.75–2.22)	1.11 (0.64–1.95)	0.24
Women						
OR^2^ (CI)	Ref	1.93 (1.41–2.62)	1.69 (1.21–2.35)	1.49 (1.05–2.11)	1.24 (0.84–1.82)	0.73
Phenolic acids (mg/day)	(< 128.8)	(128.8–170-4)	(170.4–213.5)	(213.5–277.7)	(> 277.7)	
OR^1^ (CI)	Ref	0.88 (0.70–1.13)	0.88 (0.69–1.11)	0.79 (0.62–1.01)	0.67 (0.52–0.86)	< 0.001
OR^2^ (CI)	Ref	0.90 (0.71–1.16)	0.94 (0.73–1.20)	0.84 (0.65–1.09)	0.72 (0.54–0.95)	< 0.001
Men						
OR^2^ (CI)	Ref	0.93 (0.56–1.56)	1.38 (0.85–2.24)	1.05 (0.64–1.73)	0.89 (0.54–1.46)	0.69
Women						
OR^2^ (CI)	Ref	0.91 (0.69–1.21)	0.81 (0.60–1.09)	0.78 (0.57–1.07)	0.66 (0.46–0.89)	< 0.001
Lignans (mg/day)	(< 1.33)	(1.34–1.93)	(1.94–2.73)	(2.74–4.18)	(> 4.19)	
OR^1^ (CI)	Ref	1.15 (0.89–1.48)	1.07 (0.83–1.39)	0.88 (0.67–1.10)	0.69 (0.52–0.87)	0.001
OR^2^ (CI)	Ref	1.24 (0.85–1.68)	1.11 (0.93–1.44)	0.95 (0.95–1.66)	0.78 (0.65–0.97)	0.03
Men						
OR^2^ (CI)	Ref	1.48 (0.90–2.45)	0.91 (0.54–1.56)	0.92 (0.56–1.73)	0.83 (0.48–1.45)	0.36
Women						
OR^2^ (CI)	ref	1.27 (0.93–1.74)	1.17 (0.82–1.68)	0.97 (0.63–1.48)	0.91 (0.56–1.39)	0.12

1, crude odds ratio; 2, Odds ratio adjusted for age, energy intake (continuous), gender, smoking (yes or no), alcohol consumption (yes or no), physical activity levels, BMI (continuous), and education level.

In term of total flavonoids intake, people in the upper quintile of total flavonoid dietary intake compared to the first quintile were not at a lower odd of developing NAFLD and the relationship was not significant in the crude (OR 1.26, CI 0.96–1.67; *P* = 0.47) and full adjusted model (OR 1.35, CI 0.99–1.87; *P* = 0.31). Also, in the subgroup analysis, we couldn’t find significant correlation between total flavonoids intake and NAFLD in men (OR 1.11, CI 0.64–1.95; *P* = 0.24) and women (OR 1.24, CI 0.84–1.82; *P* = 0.73).

In term of dietary phenolic acid, participants in the higher quantile of dietary phenolic acid than subjects in the reference group had a significant lower odd of NAFLD in the crude (OR 0.67, CI 0.52–0.86; *P* < 0.001) and full adjusted model (OR 0.72, CI 0.54–0.95; *P* < 0.001). Also, in the subgroup analysis, high intake of phenolics could show protective effects against NAFLD only in women (OR 0.66, CI 0.46–0.89; *P* < 0.001).

When we evaluated the association between dietary lignans intake and odds of NAFLD, it revealed that subjects in the higher quantiles of dietary lignans intake, had lower odds of NAFLD in the crude (OR 0.69, CI 0.52–0.87; *P* = 0.001) and adjusted model (OR 0.78, CI 0.65–0.97; *P* = 0.03). However, in the subgroup analysis based on gender, we couldn’t find any significant correlations.

## Discussion

The findings from the investigation indicated that participants with higher amounts of total polyphenols had a lower risk of NAFLD than subjects with lower amount of intake. Also, the results of our study showed that people who consumed a higher amount of phenolic acid and lignan through diet were less at risk of developing NAFLD than participants with low amounts of intake. However, in term of total flavonoids intake and its association with risk of NAFLD, we couldn’t find a significant correlation.

Polyphenols, widely acknowledged as water-soluble antioxidants, are present in some foods. In a case–control investigation conducted by Salehi-sahlabadi et al., encompassing 225 participants diagnosed with NAFLD and 450 control subjects, it was revealed that those in the highest tertile of total flavonoids and total phenolic acids exhibited a reduced likelihood of NAFLD in comparison to those in the lowest tertile. However, after accounting for additional confounding factors, the connection between total flavonoids and the odds of NAFLD ceased to be significant^19^. These results were in line with our findings. Also, similar to our findings, Sohouli et al., in a different case–control study, discovered that individuals in the top tertile of lignans consumption demonstrated a 66% lower likelihood of developing NAFLD in comparison to those in the lowest tertile. Hower, They did not identify any substantial association between the dietary intake of overall flavonoids and total phenolic acids with liver disease^30^. In another population-based study among the 2694 adults from the Guangzhou Nutrition and Health Study, In their research, scientists observed a gradual association between higher flavonoid intakes and a decreased likelihood of deteriorating NAFLD^31^. In our study, contrary to the findings of that particular study, there was no notable association observed between total flavonoid consumption and the likelihood of developing NAFLD.

A lack of physical activity plays a role in the development of NAFLD by leading to dysfunction in adipocytes and fostering insulin resistance. This insulin resistance, in turn, activates sterol regulatory element-binding protein-1(SREBP-1), resulting in the accumulation of fat within hepatocytes, along with elevated levels of pro-inflammatory cytokines and lipotoxicity. As a result, the activation of inflammation and fibrosis occurred due to mitochondrial dysfunction triggered by oxidative stress and the generation of reactive oxygen species^32^.Polyphenols have the potential to safeguard against NAFLD by diminishing lipid accumulation, serving as antioxidants, demonstrating anti-inflammatory properties, and enhancing insulin sensitivity^33–35^. Insulin resistance is one of the important factors in the NAFLD pathogenesis. Insulin resistance resulted from elevated levels of non-esterified fatty acids (NEFA) liberated from adipose tissues and the intermediary byproducts of hepatic lipid synthesis or breakdown^36^. Polyphenols have the capacity to increase the expression of the PPARγ gene or act as agonists for PPARγ. Consequently, this leads to a reduction in TNF-α and IL-6 levels while promoting an increase in adiponectin, phosphoenolpyruvate carboxykinase, fatty acid transport protein, and insulin receptor substrate-2. These effects collectively contribute to the enhancement of insulin resistance^37–39^. Moreover, polyphenols increased the expression of the PPARα gene or protein. PPARα, prominently present in the liver, oversees the transport of NEFA and activates enzymes associated with β-oxidation. This regulatory action restricts inflammation by impeding NF-κB and diminishing the expression of C-reactive protein^40^. It should be noticed that due to the observational nature of this study, it is not possible to confirm the cause-and-effect relationship between the consumption of polyphenols and the odds of developing NAFLD, and in our study only correlation is shown.

In the present study, we found that subjects in the higher quantiles of lignin intake had a lower odd of developing NAFLD. The primary origins of lignin consumption in our study participants were derived from various dietary sources, including wheat flour-based breads, flaxseed, legumes, garlic, almonds, olive oil, and some vegetables. These food items constitute a significant portion of a nutritious dietary pattern and are associated with a reduced risk of NAFLD^41^. In an interventional study, Yari et al. showed that flaxseed supplementation as a main source of lignin among the patients with NAFLD caused a significant improvement in the liver steatosis and inflammation^42–45^.

Based on our knowledge, the present study was the first population-based study with a big sample size that evaluated the association between total polyphenols, total flavonoids, lignin and phenolic acid with odds of NAFLD. High sample size, multi-centeredness and accurate assessment of dietary intakes were other strengths of this study. However, the present study had some limitations that should be considered. Firstly, as we mentioned, the study's observational design limits the ability to establish a cause-and-effect relationship between polyphenol consumption and the odds of developing NAFLD. The findings demonstrate correlation rather than causation. Second, the dietary intake data relied on self-reported responses obtained through an FFQ. This method may be subject to recall bias and may not accurately reflect actual dietary habits. Third, despite adjusting for several confounding variables in the regression analyses, there may still be unaccounted or residual confounding factors that could influence the observed associations. Finally, the diagnosis of NAFLD relied on medical records and reports from individual family physicians. Variability in the accuracy and consistency of these records could affect the reliability of the NAFLD diagnosis.

## Conclusion

Our findings revealed that higher intake of total polyphenols, phenolic acid, and lignin was associated with lower odds of NAFLD among adults in the Hoveyzeh cohort. This observation held true even after adjusting for potential confounding factors such as age, energy intake, gender, smoking, alcohol consumption, physical activity levels, BMI, and education level. However, no significant correlation was found between total flavonoids intake and the risk of NAFLD. Future research, including interventional studies and longitudinal investigations, is warranted to further elucidate the role of polyphenols in preventing and managing NAFLD. Nevertheless, our findings underscore the potential importance of dietary patterns rich in polyphenols as a component of strategies to mitigate the risk of NAFLD and its progression to advanced stages.

## Data Availability

The data that support the findings of this study are available from The Hoveyzeh Cohort Study (HCS), but restrictions apply to the availability of these data, which were used under license for the current study and so are not publicly available but is available with the corresponding author upon reasonable request.
